# Reduction of Thromboembolic Events in Meningioma Surgery: A Cohort Study of 724 Consecutive Patients

**DOI:** 10.1371/journal.pone.0079170

**Published:** 2013-11-14

**Authors:** Christian Valentin Eisenring, Marian Christoph Neidert, Daniel Sabanés Bové, Leonhard Held, Johannes Sarnthein, Niklaus Krayenbühl

**Affiliations:** 1 Klinik für Neurochirurgie, UniversitätsSpital Zürich, Zürich, Switzerland; 2 Institut für Sozial- und Präventivmedizin, Universität Zürich, Zürich, Switzerland; The Ohio State University Medical Center, United States of America

## Abstract

**Background:**

Meningiomas are associated with the highest postoperative rate of venous thromboembolic events (VTE) among all intracranial tumors. The aim of this study is to compare two entirely different VTE prophylaxis regimens in 724 consecutive patients undergoing meningioma surgery.

**Methods:**

Two cohorts at a single institution treated with different regimens to prevent VTE were reviewed retrospectively. Cohort A (n = 482; 314 females, mean age 57 years, range: 11–87 years) received our institutional regimen during the years 1999–2006, consisting of low-molecular-weight heparin (LMWH) and compression stockings. For cohort B (n = 242; 163 females, mean age 56.8 years, range: 16–90 years), during the years 2008–2010, the management included intraoperative 10°–20° leg elevation with intermittent pneumatic compression (IPC), heparin and LMWH administration. We compared the incidence of the endpoints pulmonary embolism (PE), deep venous thrombosis (DVT), hemorrhage and death, taking into account several known associated risk factors.

**Results:**

For all endpoints, we observed a more favorable outcome with the new regimen. The difference in incidence of PEs (cohort A: 38/482, 8%; cohort B: 6/242, 2.5%) reached statistical significance (p = 0.002). In general, patients with skull base meningiomas had a higher risk for PE (OR 2.77). Regarding VTE prophylaxis, an adjusted subgroup analysis suggests that the new regimen is particularly beneficial for patients with skull base meningiomas.

**Conclusions:**

We recommend perioperative prophylaxis using a management composed of intraoperative leg-elevation, IPC, early heparin administration and LMWH to reduce the risk for PE.

## Introduction

Meningiomas represent the second most frequent primary intracranial tumor in adults [1], with a prevalence of approximately 70.7/100 000 and an incidence of 6.0/100 000 per year in the United States (www.cbtrus.org).[2].

Venous thromboembolism (VTE), including deep vein thrombosis (DVT) and pulmonary embolism (PE), is the most common overall complication in meningioma surgery and is fatal in up to 34% of subjects.[3], [4] The VTE risk is three times higher in meningioma patients than in patients with other brain tumors, such as gliomas or brain metastases.[5], [6], [7].

The incidence of VTE in meningioma was shown to be as high as 72% using sensitive methods such as ^125^I-fibrinogen-uptake tests.[8] Several factors explaining this high incidence of VTE have been discussed in the past. The observed hypercoagulable state may be tumor induced.[6], [7], [9] Blood coagulation can be activated through the meningeal surface [1] or through damage to the vascular endothelium as a direct consequence of surgery, trauma or prior VTE.[10], [11] Brain surgery releases brain thromboplastin, altering the coagulative state.[12] Immobilization, due to intraoperative muscle relaxation, surgery of long duration or preoperative tumor-related muscle weakness of the lower extremities, results in a venous stasis that can be aggravated by postoperative bed rest.[7] Furthermore, the perioperative use of high-dose steroids as a treatment for tumor-induced vasogenic brain edema was reported as another contributing factor [12].

Preventive measures including compression stockings, early mobilization and the administration of heparin are thought to lower morbidity, but the usefulness of these measures needs quantification.

It has been shown that the prophylaxis of VTE is most effective when mechanical and pharmacological prophylaxis are combined.[7], [13], [14].

However, there is still controversy over the use of unfractionated heparin (UFH) and LMWH after meningioma resection.[1], [6], [15], [16] It is also known that only 76.2% of surgeons use mechanical DVT prophylaxis during brain tumor surgery, and LMWH was not regularly used in 72.7%.[17].

The aim of this study is to compare two entirely different perioperative management strategies in a single institution regarding safety and the prevention of VTE.

## Methods

### Study Design and Patient Population

We retrospectively analyzed all patients undergoing surgical treatment at our institution for an intracranial meningioma in the time period from 1999 to 2010. Because, in 2007,a new department chairman introduced a change in our institutional policy regarding the perioperative management to prevent VTE, we formed two cohorts - cohort A receiving the former regimen (before 2007) and cohort B receiving the novel regimen (after 2007), as depicted in Table 1.

**Table 1:** PE prophylaxis managements of cohort A and cohort B.

	Cohort A (1999–2006)	Cohort B 2008–2010
intraoperatively:	compressionstockings	IPC
		legsextended 10°–20°
post-operatively:	–	cranial CT
	–	Heparin (Liquemin)
1st postoperative day:	cranial CT	LMWH (Fragmin) untildischarge
	LMWH (Fragmin) untildischarge	

IPC = intermittent pneumatic compression, LMWH = low molecular weight heparin.

We considered and documented four clinical endpoints: hemorrhage, death, PE and DVT. The combination of PE and DVT as well as the presence of overall complication were investigated as two additional endpoints. Clinical data were obtained from the patient records at discharge and at three months after discharge.

The data were collected retrospectively, and the patients’ written consent was not obtained. The retrospective data collection without consent was approved by the local ethics committee (KEK 2011-0138, KantonaleEthikkommission Zürich).

#### Cohort A: Perioperative management 1999–2006

Cohort A included all patients undergoing craniotomy for meningioma resection during the years 1999–2006. The management consisted of the following:

1. Perioperative physical prophylaxis with compression stockings until full mobilization of the patient.
2. Administration of LMWH prophylaxis, which was given on the first postoperative day if the postoperative CT-scan (also performed on the first postoperative day) showed no significant bleeding. LMWH (Fragmin®) 5000 IE/24 h was administered subcutaneously until hospital discharge. Subjects lighter than 65 kg bodyweight were administered 2500 IE Fragmin, while subjects heavier than 80 kg bodyweight received 7500 IE.

#### Cohort B: Perioperative management 2008–2010

Cohort B was treated during the years 2008–2010. The management comprised three parts:

1. Intermittent pneumatic compression (IPC, Tyco Healthcare, KENDALL, SCD EXPRESS TM) was installed preoperatively and throughout surgery in addition to conventional compression stockings. IPC was maintained postoperatively until full mobilization.
2. The legs were elevated intraoperatively by 10°–20° to facilitate venous backflow.
3. A cranial CT control was performed within two hours after surgery to exclude hemorrhage. In the absence of bleeding complications, UFH was initiated on the day of surgery. UFH was applied continuously intravenously at 4.5 IE/kg bodyweight - the dosage was monitored under ICU surveillance. On the first postoperative day, UFH was stopped at 4 pm and replaced by 5000 IE LMWH (Fragmin®),which was administered subcutaneously at 8 pm and continued once a day until discharge. Subjects lighter than 65 kg bodyweight were administered 2500 IE LMWH/24 h, while subjects heavier than 80 kg bodyweight were given 7500 IE LMWH/24 h.

### Data Collection

The following general parameters were collected by reviewing the patient medical charts:

Age, sex, body mass index (BMI), histological grade and location of the meningioma, pre-surgical endovascular tumor embolization, duration of surgery(time interval from skin opening to skin closure), and pre-and postoperative neurological exam focusing on the presence and severity of weakness or paresis according to the Medical Research Council (MRC) Scale for muscle strength.

Hemorrhage, death, DVT and PE were checked by carefully reviewing the medical charts, including the routine 3-month postoperative follow-up notes. Any of these events counted for “overall complication”. Postoperative bleeding was evaluated by reviewing the postoperative cranial CT imaging. Hemorrhage was classified as major when it was described with the terms “rebleeding”, “fluid-level” or “large” on the radiologist’s report. The assessment of DVT and PE was not based on routine surveillance - testing for both complications was initiated in cases of clinical suspicion (DVT: swelling, pain or tenderness, warmth, redness or discoloration and distention of surface veins of an extremity; PE: new onset dyspnea, tachypnea, tachycardia, chest pain, hemodynamic instability). The diagnosis of DVT was verified by duplex sonography of the leg vessels in every case, and PE was always confirmed by multi-slice, contrast-enhanced chest CT.

We excluded ten patients lacking complete follow up: six patients who went abroad after surgery and four patients with incomplete patient records. Thus, 734 consecutive patients who were operated on between 1999 and 2010 and 724 (98.6%) were followed and included in this study.

### Statistical Methods

Statistical analyses were performed using the commercially available software IBM SPSS Statistics 20 (SPSS Inc., Chicago, IL, USA), and R version 2.15.2, (R Development Core Team, GNU General Public License). Continuous variables are presented as mean ±standard deviation (SD) and range. The patient characteristics of the two cohorts were compared using the Mann-Whitney U test or the chi-squared test, as appropriate.

For each outcome, we compared the proportion of cases in the two cohorts using odds ratios (OR), risk differences (RD), and corresponding numbers needed to treat (NNT). We also fitted logistic regression models to adjust for potential confounders using the AIC (Akaike information criterion) and the BIC (Bayesian information criterion). Both are closely related statistical criteria for model selection. Both criteria address the problem of overfitting by penalizing the number of parameters in a model. However, the BIC penalizes the number of parameters more strongly than the AIC does.

Point estimates, 95% confidence intervals (CI) and p-values are all likelihood-based. Two-tailed p values <0.05 were considered statistically significant.

First, we conducted an unadjusted comparison, without considering any of the confounders (sex, location of the tumor, WHO grade, embolization prior to surgery, paresis prior to surgery, paresis post-surgery, paresis until 3 months after discharge). Moreover, we performed a subgroup analysis for the patients with skull base meningiomas.

As a second step, we also considered potential confounders. We fitted full logistic regression models and applied backward elimination of confounders based on the AIC criterion. As an additional covariate, we considered the interaction of location (skull base meningiomas vs. non-skull base meningiomas) with the cohort indicator to assess differences in outcome parameters.

In addition, we explored whether nonlinear transformations of the continuous covariates are necessary, using fractional polynomials and splines. We did not find evidence that such transformations are necessary.

## Results

### Patient Population

#### Cohort A

Cohort A consisted of 482 patients undergoing meningioma surgery between the years 1999 and 2006, whereas in cohort B, 242 patients were operated on between 2008 and 2010.

In cohort A (n = 482; 314 females, 168 males; f:m = 1.9), the mean age was 57.0±14.6 years (range:11–87 years). The mean duration of surgery was 275.0±142.7 minutes (range: 120–1020 minutes), and the mean duration of the hospital stay was 15.1±9.6 days (range: 5–93). The average BMI was 26.0±5.1 kg/m^2^ (range 16.0–47.8 kg/m^2^).

Skull base meningiomas were treated in 181 patients (38.5%). Presurgical tumor embolization was performed in 202 subjects (41.9%). Presurgical paresis was registered in 36 (7.5%) subjects. Postsurgical paresis was observed in 52 (10.9%) and decreased to 29 patients (6.0%) 3 months after tumor resection.

#### Cohort B

In cohort B (n = 242; 163 females, 79 males; f:m = 2.1), the mean age was 56.8±14.5 years (range: 16–90 years). The mean duration of surgery was 311.4±147.5 minutes (range: 120–1015 minutes), and the mean duration of the hospital stay was 13.7±8.8 days (range: 5–62). The average BMI was 26.0±4.8 kg/m^2^ (range 17.2–44.4 kg/m^2^).

Skull base meningiomas were treated in 81 patients (40.1%). Presurgical tumor embolization was performed in 85 subjects (35.1%). Presurgical paresis was registered in 20 (8.3%), and postsurgical paresis was observed in 28 patients (11.6%) and decreased to 19 patients (7.8%) 3 months after tumor resection.

The differences in important characteristics between the two cohorts are summarized in Table 2 and 3. The only statistically significant differences between the two cohorts were a shorter hospital stay but a longer surgery duration in cohort B.

**Table 2:** Four continuous covariates (age in years, hospital stay in days, duration of surgery in minutes, body mass index) were available.

Variable	Levels	n	#NA	x	s	Min	Max
age	cohort A	482	0	57.0	14.6	11	87
	cohort B	242	0	56.8	14.5	16	90
p = 0.66	all	724	0	57.0	14.5	11	90
hospital stayin days	cohort A	482	0	14.9	9.6	5	93
	cohort B	237	5	13.6	8.8	5	62
p<0.001	all	719	5	14.5	9.4	5	93
surgery inminutes	cohort A	482	0	248.8	145.2	120	1020
	cohort B	239	3	289.4	152.4	120	1015
p<0.001	all	721	3	262.2	148.8	120	1020
BMI	cohort A	335	147	26.0	5.1	16	45
	cohort B	182	60	26.0	4.8	17	44
p = 0.79	all	517	207	26.0	5.0	16	48

The description of the continuous covariates (number of observations, number of missing observations, mean, standard deviation and range) was split into the surgery date cohorts. P-values of the Mann-Whitney test for a difference of the means between the cohorts are printed in the first column.

**Table 3:** The categorical variables are shown (sex, location of the tumor, WHO grade, embolization prior to surgery, paresis prior to surgery, paresis post-surgery, paresis until 3 months after discharge).

Variable	Levels	n_cohort A_	%_cohort A_	n_cohort B_	%_cohort B_	n_all_	%_all_
Sex	female	314	65.2	163	67.4	477	65.9
	male	168	34.9	79	32.6	247	34.1
p = 0.61	all	482	100	242	100	724	100
Location	skull base	181	38.5	81	40.1	262	39
	convexity	124	26.4	72	35.6	196	29.2
	falx	96	20.4	21	10.4	117	17.4
	intraventricular	2	0.4	1	0.5	3	0.5
	parasagittal	0	0	27	13.4	27	4
	posterior fossa	67	14.3	0	0	67	10
p<0.001	all	470	100	202	100	672	100
WHO-Grade	one	379	79.5	179	76.5	558	78.5
	two	80	16.8	47	20.1	127	17.9
	three	18	3.8	8	3.4	26	3.7
p = 0.55	all	477	100	234	100	711	100
Preoperative tumor embolization	no	280	58.1	157	64.9	437	60.4
	yes	202	41.9	85	35.1	287	39.6
p = 0.093	all	482	100	242	100	724	100
Pre-op paresis	no	443	92.5	222	91.7	665	92.2
	yes	36	7.5	20	8.3	56	7.8
p = 0.84	all	479	100	242	100	721	100
Paresis at discharge	no	427	89.1	214	88.4	641	88.9
	yes	52	10.9	28	11.6	80	11.1
p = 0.87	all	479	100	242	100	721	100
Paresis three months post-op	no	450	94	223	92.2	673	93.3
	yes	29	6	19	7.8	48	6.7
p = 0.45	all	479	100	242	100	721	100

The P-values for chi-squared tests comparing the distributions between the two surgery date cohorts are depicted in the first column.

No significant differences between the cohorts were found regarding age, sex, tumor location, WHO grade, BMI, embolization prior to surgery, paresis prior to surgery, paresis after surgery and paresis until 3 months after discharge.

### Endpoints (Unadjusted)

The outcome parameters (PE, DVT, hemorrhage, death, PE and DVT, overall complication) are given for the time period from admission until three months follow-up, unless specified otherwise - detailed results can be found in Table 4.

**Table 4:** Unadjusted results for the whole data set (change from cohort A to cohort B): Risk differences (RD) and odds ratios (OR) with 95% confidence intervals are given.

Endpoint	cohort A	cohort B	RD	CI	OR	CI	p-value	NNT
PE	8%	2.50%	−5.5%	(−8.6%, −2.2%)	0.3	(0.11, 0.66)	0.002	19
DVT	4.80%	4.60%	−0.2%	(−3.3%, 3.4%)	0.95	(0.44, 1.95)	0.9	457
Hemorrhage	6.70%	5%	−1.7%	(−5.1%, 2.1%)	0.74	(0.36, 1.42)	0.37	60
Death	1.70%	0.80%	−0.8%	(−2.5%, 1.1%)	0.49	(0.07, 1.99)	0.35	120
PE and DVT	2.70%	0.80%	−1.9%	(−3.8%, 0.2%)	0.3	(0.05, 1.1)	0.072	53
overall complication	17%	11.70%	−5.3%	(−10.4%, 0.2%)	0.65	(0.4, 1.02)	0.06	19

The p-value is a likelihood ratio p-value and thus identical for both comparison measures. The numbers needed to treat (NNT) were based on the estimated risk difference.

**Figure 1:**
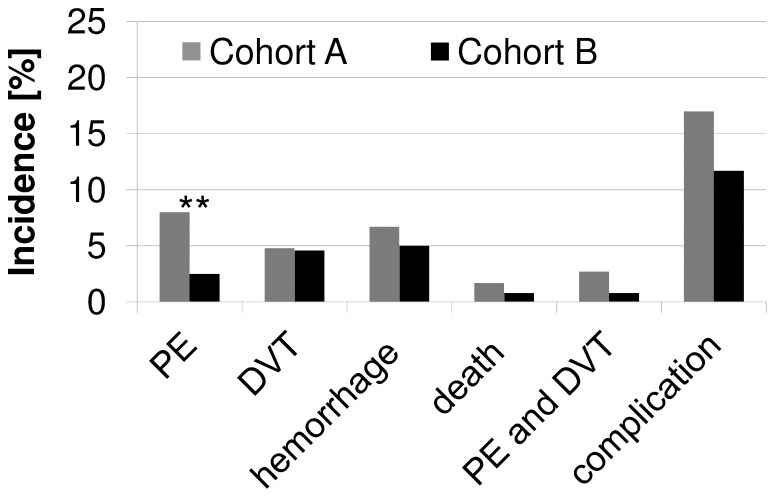
Incidence of the primary endpoints of both applied managements from admission until three months follow-up. The endpoint “PE and DVT” showed concurrent PE and DVT. “Overall complication” shows the appearance of any of the primary endpoints PE, DVT, hemorrhage or death. Bars are in % of patients in each management.

For all endpoints, a trend toward a more favorable outcome in cohort B was observed. However, a statistically significant difference between the two cohorts was observed regarding the incidence of PEs in cohort B with 2.5% (6/242) compared to cohort A with 8% (38/482) (OR 0.3; CI 0.11–0.66; p = 0.0021), as depicted in Figure 1.

### Endpoints (Adjusted)

After adjustment for potential confounders (sex, location of the tumor, WHO grade, embolization prior to surgery, paresis prior to surgery, paresis post-surgery, paresis until 3 months after exit, age in years, hospital stay in days, duration of surgery, body mass index), the OR for PE decreased further to 0.24 (CI 0.05–0.78; p = 0.02), as shown in Table 5. The OR for overall complications decreased to 0.26 (CI, 0.28–1.06; p = 0.08).

**Table 5:** Adjusted odds ratio results for changes from cohort A to cohort B (all tumor locations).

Endpoint	Odds Ratio	95% confidence interval	p-value
PE in hospital	0.17	(0.02, 0.68)	0.01
PE duringfollow up	0.24	(0.05, 0.78)	0.02
DVT in hospital	0.74	(0.19, 2.34)	0.62
DVT during follow up	0.75	(0.22, 2.18)	0.61
hemorrhage in hospital	0.55	(0.19, 1.43)	0.23
hemorrhageduring follow up	0.56	(0.19, 0.45)	0.24
death in hospital	–	–	–
death during follow up	1.37	(0.18, 7.66)	0.73
overall complication in hospital	0.51	(0.24, 1.01)	0.05
overall complication during follow up	0.56	(0.28, 1.06)	0.08

### Associated Factors

Associated factors are shown in Table 3. For the endpoint “PE”, the variables skull base location, high BMI, and hospital stay were significantly associated with a higher incidence.

Regarding the endpoint “DVT”, the variable skull base location of the tumor seemed to increase the morbidity in cohort A.

For the endpoint “hemorrhage”, a longer hospital stay and atypical meningioma (WHO grade II) were associated with an elevated risk.

For the endpoint “death”, no significant covariates were observed.

Note that for the dependent variable “PE and DVT”, the algorithm did not converge, so no results are presented.

Regarding the endpoint “overall complication”, a longer hospital stay and a skull base location were associated factors in cohort B.

It was interesting that only for the endpoints “PE” and “overall complication”, other covariates (longer hospital stay, BMI and skull base location of the tumor) were included in the best model in terms of the Bayesian information criterion (BIC).

However, the estimates and p-values should be taken with caution, as we have applied model selection.

### BMI-dependent Incidence

**Figure 2:**
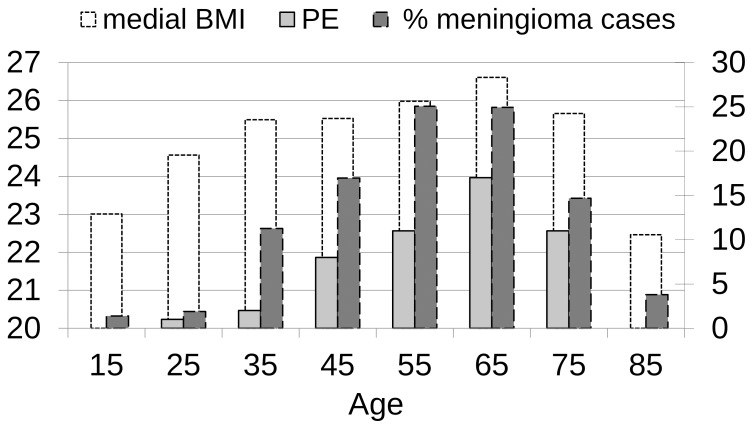
BMI with incidence of meningioma and PE. The legend for BMI is on the left side of the panel. The number of PE corresponding to each age-group and the incidence of meningioma in percent is quantified on the right side. All three factors increased with age until the sixth decade and decreased with advanced age (>70 years).

The incidence of meningioma and the incidence of a high BMI rose with age and a simultaneous increase in PE until the sixth decade in the study population, as depicted in Figure 2.

**Figure 3:**
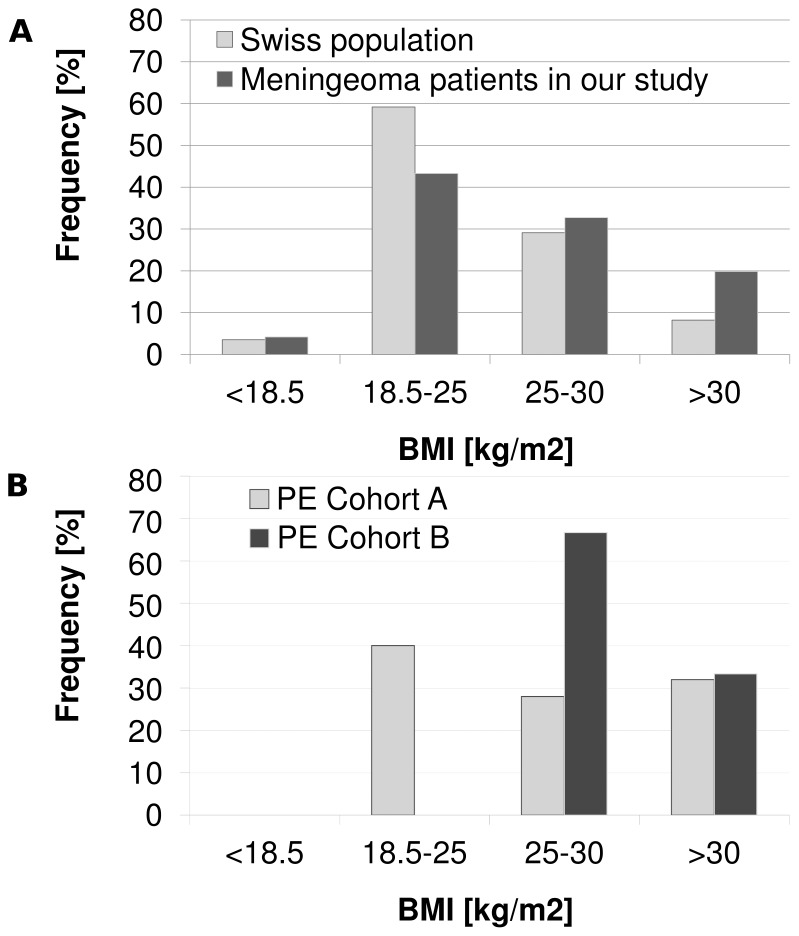
(A) Average BMI of study subjects and average national BMI. The registered BMI of subjects with a meningioma in the years 1999–2010 (n = 574) was higher than the national BMI. Shown are the proportions in weight categories based on BMI. (B) Incidence of PE assigned to BMI-categories. The distribution of patients with PE, depicted for BMI categories of the two cohorts separately.

The BMI of the study population was compared with the BMI of the general population of Switzerland (www.bfs.admin.ch) older than 15 years in 2007. Figure 3 shows that the group of the overweight and obese is disproportionately overrepresented in our study population and that normal weight patients (BMI 18.5–25) benefited most from the new regimen (cohort B).

### Subgroup Analyses

#### PE in skull base meningiomas

**Figure 4:**
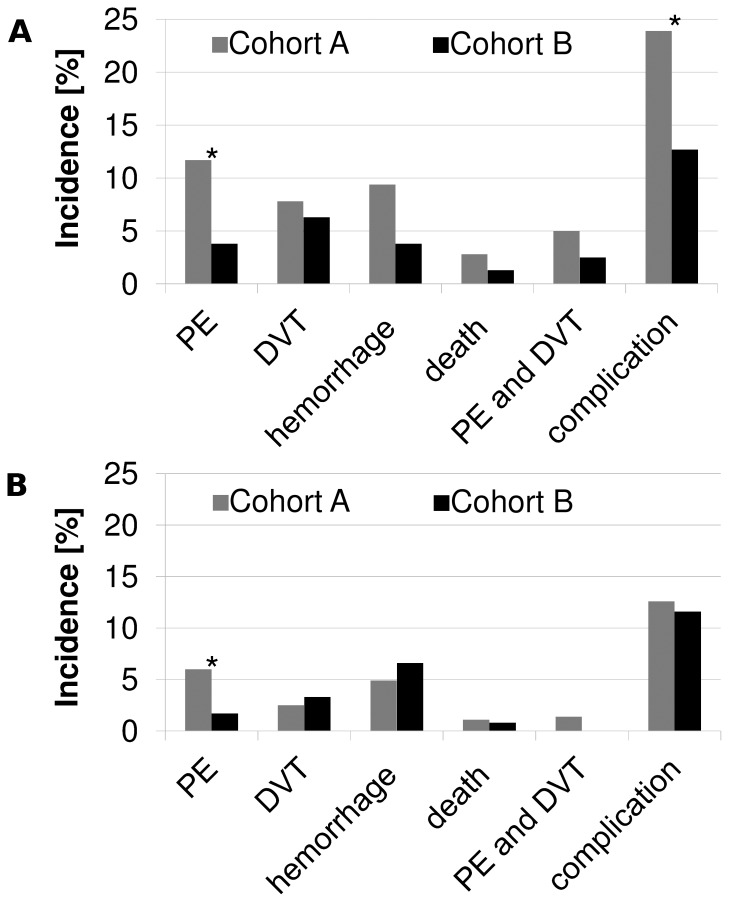
Comparison between meningioma locations. Cases with a skull base location (A) had a higher risk for PE (OR: 2.77 with 95% CI 1.15 to 7.07; p = 0.0096) compared to subjects with non-skull base meningioma. (B). The difference of odds ratio (OR) estimates between skull base and non-skull base meningioma is significant for DVT (p = 0.02) and overall complication (p = 0.05).

A total of 262 out of 724 patients (39%) were treated for a skull base meningioma (cohort A: 181/479, 38.5%; cohort B: 81/242, 40.1%). Patients with skull base meningiomas showed a higher rate of PE in general, as depicted in Figure 4. Cases with a skull base location had a higher risk for PE (OR: 2.77 with 95% CI 1.15 to 7.07; p = 0.0096) compared to subjects without skull base meningioma. In cohort A, 11.7% of the patients with skull base meningioma suffered a PE by 3 months postoperative follow-up, whereas in cohort B, only 3.8% had a PE (unadjusted OR: 0.3; CI 0.07–0.9; p = 0.03; NNT 13).

#### PE in non-skull base meningiomas

In analogy to the skull base meningioma group, the subgroup of patients with non-skull base lesions also benefited from the novel anti-thrombotic regimen (cohort B), but to a lesser degree. In the group of non-skull base meningiomas, the rate of PE was 6% in cohort A and 1.7% in cohort B, as shown in Figure 4. (Unadjusted OR: 0.26; CI 0.04–0.94; p = 0.04; NNT 24).

#### PE in skull base meningiomas vs. non-skull base meningiomas

When comparing the benefit of the new regimen (cohort B) between the two subgroups (skull base meningiomas vs. non-skull base meningiomas) and when additionally adjusting for the covariates mentioned above, there is a statistically significant larger benefit for the subgroup of skull base meningiomas from the novel anti-thrombotic regimen. The adjusted odds ratio (adjusted OR 0.09 for change from cohort A to cohort B) is significantly smaller in the skull base group than in the non-skull base group (adjusted OR 0.71), p = 0.02 as shown in Table 6.

**Table 6:** Adjusted odds ratio results (change from cohort A to cohort B).

Endpoint	OR (sb)	CI	OR (non-sb)	CI	P (diff)	P (drop)
Hemorrhage 3 m f/u	0.25	(0.03,1.01)	1.2	(0.29,4.16)	0.12	0.15
death 3 m f/u	0.88	(0.04,7.28)	3.11	(0.11,93.37)	0.50	0.75
PE 3 m f/u	0.09	(0,0.53)	0.71	(0.01,3.43)	0.12	0.02
DVT 3 m f/u	0.28	(0.04,1.18)	4.2	(0.64,34.57)	0.03	0.07
Complication 3 m f/u	0.26	(0.09,0.67)	1.31	(0.5,3.35)	0.02	0.01

Shown are the odds ratio (OR) estimates with corresponding 95% confidence intervals (CIs) for patients with skull base location (sb) and other locations (non-sb) of the tumor after 3 months of follow-up (3 m f/u). The difference between the two location groups was tested, and the p-value is reported (diff);additionally, the effect of excluding the exposure variable was tested, giving the p-value in the right-most column (drop).

## Discussion

Venous thromboembolic complications are a major contributor to morbidity following meningioma surgery and can be reduced by the use of standardized protocols.[18].

In addition, the removal of meningiomas is frequently associated with excessive intraoperative bleeding and an elevated risk for postoperative intracranial hemorrhage. The rich vasculature of meningioma is a contributing factor for local hemorrhages.[4], [5] Both postoperative hemorrhagic complications and VTE mainly occur within the first 2 postoperative weeks after meningioma resection[13], [19]. Postoperative management in meningioma surgery is therefore a balancing act between risking venous thromboembolic events and risking hemorrhage. There is a commonly agreed upon optimal strategy to prevent VTE using a combined approach including physical and pharmacological therapy.[18] IPC decreases the incidence of VTE by 50% and is the preferred prophylaxis in neurosurgery.[17] Several studies have shown that the combination of LMWH and compression stockings is as safe as and more efficacious than compression stockings alone.[6], [17], [18], [20], [21].

### Alternative Prophylactic Regimens

In one study by Gerber et al., the VTE prophylaxis protocol consisted of mechanical prophylaxis with preoperative TED compression stockings and intermittent pneumatic compression. Pharmacological prophylaxis with unfractionated heparin (5000 units subcutaneously) was started 24 hours postoperatively and administered twice daily. Early postoperative ambulation was started on the first postoperative day.[1].

Another DVT prophylaxis protocol was to administer elastic stockings pre- and postoperatively, starting from the day of hospitalization until the subjects were completely mobile. Mechanical graduate pneumatic sequential leg compression was added perioperatively. Sodium tinzaparin (LMWH) was started 24 hours postoperatively, and in high-risk patients, tinzaparin was administered regularly 5 to 7 days before surgery and stopped on the day before surgery. After surgery, the application of tinzaparin continued until the subjects were completely mobile. With high-risk patients, tinzaparin was continued for at least 3 weeks postoperatively.[15].

A third study administered enoxaparin (a LMWH) starting between 24 h and 48 h as a subcutaneous injection after the resection of an intracranial meningioma. The treatment lasted between 1 and 7 days, usually with a dose of 40 mg daily. The use of post-operative physical prophylaxis with TED stockings or pneumatic sequential compression was also noted. [6] The rate of both PE and DVT was 0% with enoxaparin. In the non-enoxaparin group, the incidence of PE was 3.2%, and the incidence of DVT was 4.8%. Hemorrhage occurred in 12.5% of the enoxaparin-treated subjects and in 12.9% with the non-enoxaparin treatment. These results suggest no increased risk of postoperative hemorrhage after administration of LMWH. The ACCP Guidelines recommend, for major neurosurgery patients, routine thrombosis prophylaxis with the use of IPC or postoperative LMWH or UFH. [22].

The routine administration of LMWH within the first 48 h after the surgical resection of meningiomas seems to decrease the incidence of postoperative thromboembolic events without increased risk for bleeding complications.

### Pulmonary Embolism

**Figure 5:**
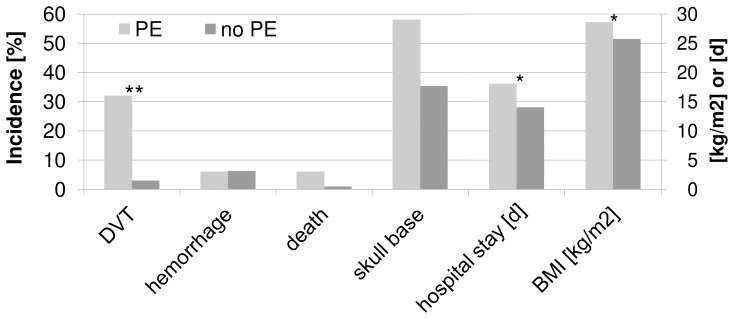
PE associated factors. The comparison of cases with PE and cases without PE showed that PE is significantly associated with skull base meningioma (p = 0.01), prolonged hospital stay (p = 0.01 difference = 2.87 days) and higher BMI (p = 0.03 difference = 4.05 kg/m^2^).

PE during the hospital stay decreased from 7.3% in cohort A to 1.7% in cohort B (p = 0.00055), and it decreased from 8% to 2.5% (p = 0.0021) for the period from admission until a three-month postoperative follow-up. This fact highlights the observation that the majority of PEs occurred during hospitalization. For the PE endpoint, a skull base location, a higher BMI and a longer hospital stay are associated with a higher risk, as depicted in Figure 5.

Independent risk factors associated with the development of DVT or PE included older age (P<0.0001), paresis (0.002), large tumor size and a duration of surgery longer than 4 h.[23].

A longer hospital stay could be both a risk factor (immobilization) and a consequence of PE. Our data do not allow that discrimination. The decrease in PE is most likely due to the earlier administration of heparin, perioperative IPC and extended intraoperative leg positioning. The large decrease in PE in cohort B was unexpected in relation to the stable rates of DVT in both cohorts.

It is also interesting to note that the rate for DVT in cohort A is lower than the rate of PE. This observation is counterintuitive, as the common pathomechanism is a PE as a complication of a DVT. However, the two diagnoses are made with different diagnostic modalities, harboring a different sensitivity. PE is diagnosed with a very sensitive method (contrast-enhanced spiral CT of the thorax), and a diagnosis of DVT was not sought in all PE cases, as PE and DVT end in a common therapeutic pathway (anticoagulation).

### Postoperative Hemorrhage

Postoperative hemorrhage following neurosurgical procedures in patients receiving prophylactic heparin is reported to occur in 6% of meningioma procedures compared to 3.2% after other major intracranial interventions.[14] In addition to tumor-specific high vascularization, the intensity of the prophylactic anticoagulation effect is most likely an important risk factor contributing to intracerebral hemorrhage.[24], [25] Any effective treatment that is associated with a lower risk of bleeding would be useful.[20], [26]The incidence of postoperative hemorrhages decreased from 6.7% in cohort A to 5% in cohort B. These rates of hemorrhage are in accordance with the literature mentioned above. This decrease was initially unexpected considering that the earlier initiation of heparin administration (cohort B) should be associated with a higher risk for hemorrhage. We assume that the early postoperative CT scan in cohort B missed some postoperative (clinically unapparent) bleedings that would have been detected with a later CT scan, as administered in cohort A. In all cases of significant postoperative hemorrhage, heparin administration was delayed.

### Skull Base Meningiomas

On one hand, skull base meningiomas were associated with the highest rate of VTE in our study; on the other hand, these patients showed the most pronounced benefit with an improved prophylactic regimen (cohort B). Resections at the skull base frequently require a long surgery duration (1 hour 24 minutes (37%) longer than non-skull base lesions in our series).

Intraoperative hemostasis might be one factor contributing to this higher incidence of VTE. In addition, the neurological outcome is less favorable with skull base meningiomas.[27], [28], [29]A skull base meningioma can enclose cranial nerves, the pituitary stalk or vascular structures, and complete resections are both rare and associated with a high morbidity.[29], [30] Therefore, postoperative immobilization and blood stasis are increased in this subgroup of meningiomas. Our data agree with research findings that described a higher susceptibility to thromboembolic complications in subjects suffering from meningioma located at the skull base.[8], [31], [32] Interestingly, the average operation duration in skull base tumors increased from 5 hours 3 minutes in cohort A to 6 hours 1 minute in cohort B, indicating that the lower rate of VTE in cohort B cannot be explained by a shorter surgery duration and is more likely an effect of the new regimen.

### Limitations of the Study

A rigorous comparison between two prophylactic regimens should, of course, be performed in a prospective randomized controlled trial. In our retrospective study, the subjects were recruited over a period of eleven years and, in principle, several aspects of the treatment might have changed in this period. The improved outcome in cohort B could thus be regarded as progress-related bias. However, progress-related bias was not present for surgical technique, as no major progress occurred with respect to meningioma treatment, and the approaches for rehabilitation have also remained the same.

Regarding the detection of clinical symptoms, different treating physicians might have had different thresholds for ordering a diagnostic work-up regarding thromboembolic complications. Additionally, smoking habits may have changed over time, and the smoking status of the included patients was not assessed. Smoking is a known risk factor for developing thromboembolic events. However, it seems unlikely that a systematic difference in these aspects could be relevant to the difference between the two cohorts or between skull base and non-skull base meningioma patients.

To assess the neurological impact of the postoperative bleeding, the subjects with postoperative hemorrhage would have to be further stratified with respect to clot volume or the need for surgical evacuation. While we assume that the rate of severe hemorrhages has also decreased, the limited information available from patient charts constrains us to report the reduction in CT-findings between the two cohorts.

## Conclusions

The introduction of the early postoperative application of UFH, intraoperative leg extension and the application of IPC in addition to LMWH and regular compression stockings significantly reduced the rate of PE after meningioma surgery. The patients with skull base meningiomas benefited the most from this management.

The earlier administration of heparin (UFH) did not result in increased complications from postoperative hemorrhage. We recommend the management applied in cohort B, in particular for patients with skull base meningiomas, to prevent perioperative venous thromboembolic events.

## References

[1] Gerber DE, Segal JB, Salhotra A, Olivi A, Grossman SA, et al (2007) Venous thromboembolism occurs infrequently in meningioma patients receiving combined modality prophylaxis. Cancer 109: 300–305. 17154163 10.1002/cncr.22405

[2] Porter KR, McCarthy BJ, Freels S, Kim Y, Davis FG (2010) Prevalence estimates for primary brain tumors in the United States by age, gender, behavior, and histology. Neuro Oncol 12: 520–527. 20511189 10.1093/neuonc/nop066 PMC2940648

[3] Caroli M, Locatelli M, Prada F, Beretta F, Martinelli-Boneschi F, et al (2005) Surgery for intracranial meningiomas in the elderly: a clinical-radiological grading system as a predictor of outcome. J Neurosurg 102: 290–294. 15739557 10.3171/jns.2005.102.2.0290

[4] Sawaya R, Decourteen-Meyers G, Copeland B (1984) Massive preoperative pulmonary embolism and suprasellar brain tumor: case report and review of the literature. Neurosurgery 15: 566–571. 6493467 10.1227/00006123-198410000-00019

[5] Al-Mefty O (1991) MENINGIOMAS. XXI+630P p.

[6] Cage TA, Lamborn KR, Ware ML, Frankfurt A, Chakalian L, et al (2009) Adjuvant enoxaparin therapy may decrease the incidence of postoperative thrombotic events though does not increase the incidence of postoperative intracranial hemorrhage in patients with meningiomas. Journal of Neuro-Oncology 93: 151–156. 19430892 10.1007/s11060-009-9886-4

[7] Koray Özduman RF (2010) Meningiomas: A Comprehensive Text, Perioperative management of patients with meningiomas. In: M. Necmettin Pamir PMB, Rudolf Fahlbusch, editor. Acta Neurochirurgica. 800.

[8] Sawaya R, Zuccarello M, Elkalliny M (1989) Brain-Tumors and Thromboembolism - Clinical, Hemostatic, and Biochemical Correlations. Journal of Neurosurgery 70: A314–A314.

[9] Whittle IR, Smith C, Navoo P, Collie D (2004) Meningiomas. Lancet 363: 1535–1543. 15135603 10.1016/S0140-6736(04)16153-9

[10] Keller E (2011) Kapitel 20 Thromboembolieprophylaxe. In: Schwab S, Schellinger P, Werner C, Unterberg A, Hacke W, editors. NeuroIntensiv: Springer. 810.

[11] Samandouras G (2010) The neurosurgeon's handbook. Oxford: Oxford University Press. xxix, 930 p., [912] p. of plates p.

[12] Greenberg MSMD (2010) Handbook of neurosurgery. New York: Thieme. xiv, 1337 p. p.

[13] Gerlach R, Krause M, Seifert V, Goerlinger K (2009) Hemostatic and hemorrhagic problems in neurosurgical patients. Acta Neurochirurgica 151: 873–900. 19557305 10.1007/s00701-009-0409-z

[14] Gerlach R, Scheuer T, Beck J, Woszczyk A, Seifert V, et al.. (2003) Risk of postoperative hemorrhage after intracranial surgery after early nadroparin administration: results of a prospective study. Neurosurgery 53: 1028–1034; discussion 1034–1025. 10.1227/01.neu.0000088565.15719.22 14580268

[15] Chibbaro S, Tacconi L (2008) Safety of deep venous thrombosis prophylaxis with low-molecular-weight heparin in brain surgery. Prospective study on 746 patients. Surgical Neurology 70: 117–121. 18262633 10.1016/j.surneu.2007.06.081

[16] Raslan AM, Fields JD, Bhardwaj A (2010) Prophylaxis for venous thrombo-embolism in neurocritical care: a critical appraisal. Neurocrit Care 12: 297–309. 20033354 10.1007/s12028-009-9316-7

[17] Carman TL, Kanner AA, Barnett GH, Deitcher SR (2003) Prevention of thromboembolism after neurosurgery for brain and spinal tumors. Southern Medical Journal 96: 17–22. 12602707 10.1097/01.SMJ.0000047628.44490.B2

[18] Wong JM, Panchmatia JR, Ziewacz JE, Bader AM, Dunn IF, et al (2012) Patterns in neurosurgical adverse events: intracranial neoplasm surgery. Neurosurg Focus 33: E16. 10.3171/2012.7.FOCUS12183 23116096

[19] Wiemels J, Wrensch M, Claus EB (2010) Epidemiology and etiology of meningioma. J Neurooncol 99: 307–314. 20821343 10.1007/s11060-010-0386-3 PMC2945461

[20] Agnelli G (1999) Prevention of venous thromboembolism after neurosurgery. Thrombosis and Haemostasis 82: 925–930. 10605805

[21] Kleindienst A, Harvey HB, Mater E, Bronst J, Flack J, et al (2003) Early antithrombotic prophylaxis with low molecular weight heparin in neurosurgery. Acta Neurochirurgica 145: 1085–1091. 14663565 10.1007/s00701-003-0142-y

[22] Geerts WH, Bergqvist D, Pineo GF, Heit JA, Samama CM, et al (2008) Prevention of venous thromboembolism: American College of Chest Physicians Evidence-Based Clinical Practice Guidelines (8th Edition). Chest 133: 381S–453S. 18574271 10.1378/chest.08-0656

[23] Chaichana KL, Pendleton C, Jackson C, Martinez-Gutierrez JC, Diaz-Stransky A, et al (2013) Deep venous thrombosis and pulmonary embolisms in adult patients undergoing craniotomy for brain tumors. Neurol Res 35: 206–211. 23336127 10.1179/1743132812Y.0000000126 PMC3991124

[24] Schulman S, Beyth RJ, Kearon C, Levine MN, Physicians ACoC (2008) Hemorrhagic complications of anticoagulant and thrombolytic treatment: American College of Chest Physicians Evidence-Based Clinical Practice Guidelines (8th Edition). Chest 133: 257S–298S. 18574268 10.1378/chest.08-0674

[25] Raabe A, Gerlach R, Zimmermann M, Seifert V (2000) Practice of perioperative thromboembolic prophylaxis in neurosurgery: results of a German survey]. Zentralblatt für Neurochirurgie 61: 103. 10986759 10.1055/s-2000-8267

[26] Macdonald RL, Amidei C, Lin G, Munshi I, Baron J, et al.. (1999) Safety of perioperative subcutaneous heparin for prophylaxis of venous thromboembolism in patients undergoing craniotomy. Neurosurgery 45: 245–251; discussion 251–242. 10.1097/00006123-199908000-00008 10449068

[27] Black P, Morokoff A, Zauberman J, Claus E, Carroll R (2007) Meningiomas: science and surgery. Clin Neurosurg 54: 91–99. 18504903

[28] Riffaud L, Mazzon A, Haegelen C, Hamlat A, Morandi X (2007) Surgery for intracranial meningiomas in patients older than 80 years. Presse Medicale 36: 197–202. 10.1016/j.lpm.2006.11.009 17259027

[29] Roser F, Ebner FH, Ritz R, Samii M, Tatagiba MS, et al (2007) Management of skull based meningiomas in the elderly patient. Journal of Clinical Neuroscience 14: 224–228. 17258130 10.1016/j.jocn.2005.12.004

[30] Stienen MN, Lucke S, Fournier JY, Hildebrandt G, Gautschi OP (2010) [The intracranial meningioma - therapeutic possibilities and limitations]. Praxis (Bern 1994) 99: 1479–1494. 21125533 10.1024/1661-8157/a000321

[31] Black P, Kathiresan S, Chung W (1998) Meningioma surgery in the elderly: A case-control study assessing morbidity and mortality. Acta Neurochirurgica 140: 1013–+. 10.1007/s007010050209 9856244

[32] Chen CM, Huang AP, Kuo LT, Tu YK (2011) Contemporary surgical outcome for skull base meningiomas. Neurosurg Rev 34: 281–296; discussion 296. 10.1007/s10143-011-0321-x 21614426

